# Records of the non‐native alga *Acanthophoraspicifera* (Rhodophyta) and their colonial epibionts in La Paz Bay, Gulf of California

**DOI:** 10.3897/BDJ.11.e114262

**Published:** 2023-11-20

**Authors:** María A. Mendoza-Becerril, Francisco F. Pedroche, Mariae C. Estrada-González, Elisa Serviere-Zaragoza

**Affiliations:** 1 El Colegio de la Frontera Sur (ECOSUR), Chetumal, Mexico El Colegio de la Frontera Sur (ECOSUR) Chetumal Mexico; 2 Departamento de Ciencias Ambientales, Universidad Autónoma Metropolitana, Unidad Lerma, Estado de Mexico, Mexico Departamento de Ciencias Ambientales, Universidad Autónoma Metropolitana, Unidad Lerma Estado de Mexico Mexico; 3 Medusozoa México, La Paz, Mexico Medusozoa México La Paz Mexico; 4 Centro de Investigaciones Biológicas del Noroeste (CIBNOR), La Paz, Mexico Centro de Investigaciones Biológicas del Noroeste (CIBNOR) La Paz Mexico

**Keywords:** red algae, Bryozoa, Hydrozoa, Pacific Ocean, Mexico

## Abstract

*Acanthophoraspicifera*, a red alga considered an alien species, was discovered for the first time on the Pacific coast of Mexico in 2006 from a locality inside La Paz Bay, Gulf of California. Since then, more records have shown its presence, 17 localities having been added up to 2015. A two-year field study (2020-2022) visiting 31 sites along the coast of La Paz Bay, complemented with data from literature and citizen science, resulted in a database of 709 entries that spans the data from 2004 to 2023. These data showed a distribution that goes from Punta Coyote, close to Boca Grande, the northern entrance to the Bay to Playa Tecolote in the south, more than 100 km of coastline, including Espiritu Santo Archipelago, an area considered a natural reserve since 2007. Anthropogenic activity and environmental variables did not present statistical differences that explain *A.spicifera* spreading. It represents a naturalised alien species without evidence of a negative impact. Still, it soon could acquire the status of invasive species together with its epibionts Bryozoa and Hydrozoa detected in this study.

## Introduction

The marine erect sea moss *Acanthophoraspicifera* (M. Vahl) Børgesen belongs to the family Rhodomelaceae (Ceramiales, Rhodophyta). It was described from St Croix West Indies as *Fucusspiciferus* M.Vahl, 1802 (Vahl 1802). Since then, its western Atlantic distribution has gone from Florida to northern Brazil and Caribbean islands (Guiry and Guiry 2023) with records in the mainland coast of the Gulf of Mexico from Veracruz to Quintana Roo (García García et al. 2020). Considered by some authors as a pantropical species (De Jong et al. 1999), this name has been recorded for areas outside this range, for example, Argentina and China, amongst others (Guiry and Guiry 2023). This red alga is sparingly branched, lacking spines; if they are present, they are only in meagre numbers or solitary on main axes. On indeterminate branches, spines are crowded and smaller towards the apex and those on the branchlets are mostly grouped at the apices (De Jong et al. 1999). *Acanthophoraspicifera* has been considered an alien species in the Pacific Ocean since its discovery in 1952 on Hawaii’s coasts, where it was introduced throughout the West (Doty 1961), particularly in Manila, Philippines where it has a cryptogenic status (Tsuda et al. 2008) and, based on O'Doherty and Sherwood (2007), not possible to infer if it was introduced from the Western Pacific or Southern Pacific due to genetic similarity amongst distant sites. However, it is originally native to the Tropical Atlantic (De Jong et al. 1999).

Dawson, a well-known seaweed collector, did not cite or house specimens of the genus from the Pacific coast of Mexico during 1940-1966 (Dawson 1944, Dawson 1962, Dawson 1966), as well as the floristic updates carried out up to July 1991 in the same marine zone (González-González et al. 1996, Riosmena-Rodríguez and Paul-Chávez 1997). The first published record was from La Paz (south-western Gulf of California) (Muñoz-Ochoa et al. 2010). However, Ávila et al. (2012) cited material not published before the summer of 2006, growing in a small area in the locality of Costa Baja, near the Port of Pichilingue (Águila-Ramírez et al. 2012, Aguilar-Rosas et al. 2014). Norris (2014) did not mention this taxon in his global study of seaweeds from the northern Gulf of California. Later, it was mentioned only for the site Playa El Caimancito, close to La Paz, as the known distribution for this species (Norris et al. 2017). However, the species has been reported in other biological or ecological studies (Riosmena-Rodríguez et al. 2014, Schnoller et al. 2016,Antonio-Robles et al. 2021) or others related to its chemical composition or biological activity (Muñoz-Ochoa et al. 2010, Tenorio-Rodriguez et al. 2017, Di Filippo-Herrera et al. 2019, Schnoller et al. 2020). One of the most evident effects of its introduction to La Paz Bay is substratum monopolisation, as this alien seaweed forms dense beds colonising almost all types of hard substrata from the intertidal to 5-6 m depth, such as rocks, mussels, sponges and coral rubble (Ávila et al. 2012). The success of this species in occupying new habitats has been related to its high morphological plasticity, sexual and asexual reproductive strategies, adaptability to a wide range of hydrological conditions and its successful epiphytism on other algae (Russell 1992).

Worldwide, *A.spicifera* has been recorded as a frequent substrate for fouling organisms such as hydroids (Migotto 1996, Chiaverini 2002, Oliveira and Marques 2007, Ávila et al. 2012), as well as sponges and seaweeds in records from La Paz Bay (Ávila et al. 2012, Méndez-Trejo et al. 2014). Likewise, non-fouling organisms, such as annelids, arthropods, echinoderms and molluscs, use this macroalga as refugia (Méndez-Trejo et al. 2014), showing that *A.spicifera* could be an excellent hitchhiking medium for travelling along the seas. For this reason, the research on non-native species should also focus on whether they have epibiont growth, particularly epibionts with a sessile habit because fouling and rafting on substrata are common dispersal mechanisms for these (Winston 2012, Kuhlenkamp and Kind 2013, Gutow et al. 2015) and some have been related to potential changes in the ecosystem, as mentioned for colonial encrusting epibionts as bryozoans and hydrozoans (Yorke and Metaxas 2011, Carlton et al. 2019). The goals of this paper are to present the updated distribution of *A.spicifera* and their colonial encrusting Bryozoa and Hydrozoa, as well as to explore some environmental data and information on anthropogenic activity, by trying to correlate the matrices of distribution, environmental data and anthropogenic activity for the inner region of a subtropical bay of the Gulf of California, La Paz Bay.

## Material and methods

### Search of published literature and citizen-science data

**Figure 1.**
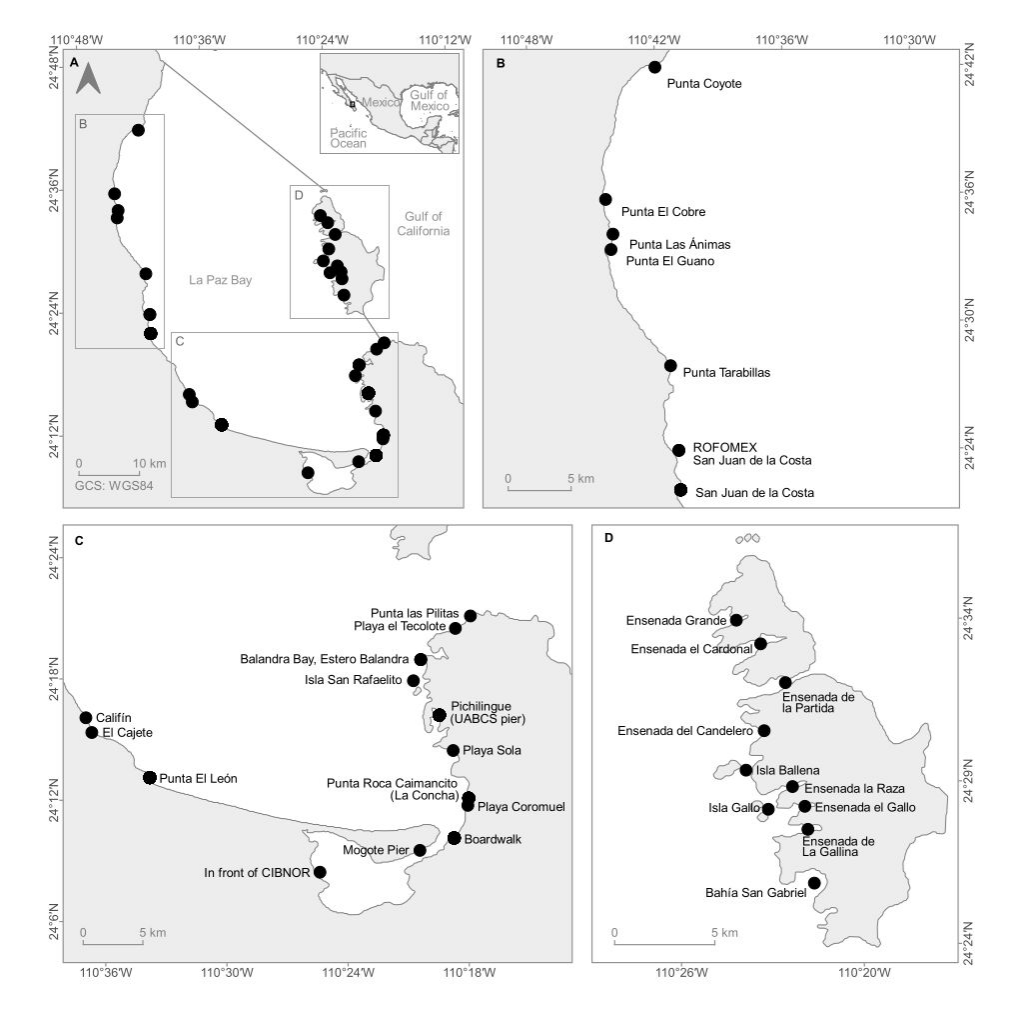
Sites visited to detect, observe and collect *Acanthophoraspicifera* during 2020-2022 from La Paz Bay, Baja California Sur, Mexico: **A** La Paz Bay; **B** Northern section; **C** Southern section; **D** Espiritu Santo Archipelago.

This study was conducted in La Paz Bay, Baja California Sur, Gulf of California, a natural anchorage of 1200 km^2^ (González-Acosta et al. 2018). For this study, La Paz Bay limits are Punta Coyote (24°41'52"N, 110°41'57"W) on the northern coast and Punta Pilitas (24°21'07"N, 110°17'56"W) in the southeast, with a coastline of almost 134 km (Fig. 1). There is an Archipelago, Espíritu Santo (ESA), with two islands, Espíritu Santo and La Partida. It has an arid climate with scarce and irregular precipitation (180 mm yr^–1^), a rate of evaporation of 215 mm yr^–1^ and the tides are predominantly mixed semi-diurnal (Obeso-Nieblas et al. 2004). Two seasons are clearly distinguished during the year: a cold season with an average temperature range of 20.5-26.0°C from December-May and a warm season of 26.0-31.0°C from June-November (Guevara-Guillén et al. 2015).

This study included data from three sources (literature, citizen science and fieldwork) to integrate and update *A.spicifera* information. The literature search included only papers cited in Google Scholar (https://scholar.google.com/) databases published until 2023, using the following search terms: “*Acanthophoraspicifera* La Paz Bay”, “*Acanthophoraspicifera* Hydrozoa La Paz Bay” and “*Acanthophoraspicifera* Bryozoa La Paz Bay”, excluding duplicate literature results and including only scientific articles with records of hydrozoans and bryozoans growing on *A.spicifera* and records of only *A.spicifera*.

The search term for citizen-science data (Naturalista 2023) was “*Acanthophoraspicifera*”. Only records with photos from La Paz Bay and marked as “degree of research” on the platform Naturalista were included. As it is complicated to locate the presence of epibionts in the photos, only the macroalga records were considered.

### Field and lab work

**Figure 2.**
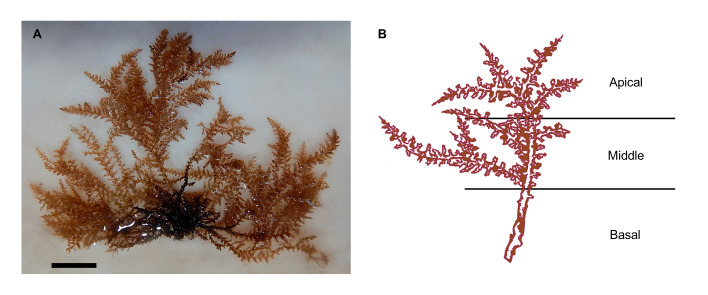
*Acanthophoraspicifera*: **A** Specimen from Punta Roca Caimancito; **B** Sections of the thallus where epibionts were detected. Scale bar: A, 2 cm.

Along La Paz Bay, including ESA, 31 sites were visited looking for the presence of *A.spicifera* for three years (2020-2022), including the places cited in scientific papers (Fig. 1). In six sites (San Juan de la Costa, Punta El León, In front of CIBNOR, Boardwalk, Punta Roca Caimancito and Pichilingue), a total of 31 thalli were collected manually by the same team and with the help of a knife or through scraping on substrates (e.g. sand, rocks). More thalli were collected at sites where thalli were present with more than 40 thalli. All thalli collected were fixed in 96% ethanol for epibionts' observed presence or absence. In the remaining 25 sites, only the presence or absence of thalli was observed by snorkelling or scuba diving (Field observations = FO; Fig. 1). The depth, temperature and salinity were measured *in situ*. *Acanthophoraspicifera* specimens were identified according to De Jong et al. (1999). The thalli were examined in the laboratory and in each thallus, sessile colonial epibionts (Hydrozoa or Bryozoa) were recorded using the microscopes Zeiss Stemi 2000-C and Zeiss Axio Scope A1. Each thallus was segmented into three parts: the basal segment consisted of the first third closest to the disc and part of the stem, the middle segment included the central part of the alga and the last third of the thallus from the middle part to the tips of the alga was catalogued as the apical segment (Fig. 2). Epibionts were identified using morphological criteria according to Brusca (1980). The nomenclature used here follows WoRMS Editorial Board (2023). After their analyses, specimens were deposited in the Macroalgae Laboratory, Centro de Investigaciones Biológicas del Noroeste, S.C. (algae and epibionts) and in the Herbarium FBCS of the Universidad Autónoma de Baja California Sur under the code FBCS20274 (algae).

### Sites' environmental data

The environmental satellite variables of chlorophyll-*a* (mg m^-3^; Chlo-*a*), Particulate Inorganic Carbon (mol m^-3^; PIC), Particulate Organic Carbon (mg m^-3^; POC), Photosynthetically Available Radiation (Einstein m^-2^ d^-1^; PAR) and Sea Surface Temperature (°C; SST) were obtained from Aqua MODIS-Aqua Level-3 products, with a daily time scale and a spatial resolution of 4 km (NASA Goddard Space Flight Center 2023). For each site and date (referred to as points), variable values were extracted using Quantum GIS (QGIS) v.3.16.16 (QGIS Development Team 2023). When the points had null data, interpolation on the raster imagery edge was estimated, based on the QGIS tool "Fill no data," which uses inverse distance weighting, with ten pixels as the maximum distance.

### Database

A database was built with ecological and environmental information on *A.spicifera* in La Paz Bay from 2004-2023. This database contains literature, citizen science and field data: presence and size of the macroalga, segment of the thallus with epibionts (Hydrozoa or Bryozoa), presence of Hydrozoa, Bryozoa or both, sampling data (year, month, day), place and coordinates of collection or recording, depth, environmental variables *in situ* (salinity sea surface, temperature), environmental satellite variables Chlo-*a*, PAR, PIC, POC and SST and anthropogenic activities in the site, such as natural protected area, tourism and local uses, fishing, nutrient supply, metal supply, nautical traffic, runoff water, mining, physical habitat modification, dump, industry and mangrove deforestation (Méndez et al. 2006, Velasco García 2009, Mendoza-Salgado et al. 2011, Ávila et al. 2012, Secretaría de Medio Ambiente y Recursos Naturales and Comisión Nacional de Áreas Naturales Protegidas 2014, Tovar Lee et al. 2015, Secretaría de Medio Ambiente y Recursos Naturales and Recursos Naturales and Comisión Nacional de Áreas Naturales Protegidas 2016, Roldán-Wong et al. 2020, Mendoza-Becerril et al. 2022, Estrada-González pers. obs.). This database is available in Mendoza-Becerril et al. (2023).

### Spatial analysis

Maps with the spatial attributes of *A.spicifera* by site, such as data source, current presence and absence of this macroalga, as well as the expansion of its records over time and the records of Bryozoa and Hydrozoa colonial epibionts over time, were elaborated using Quantum GIS (QGIS) v.3.16.16 (QGIS Development Team 2023). The sites are represented by a point and a number in the maps; this number accompanies each site name mentioned in the results and discussion as (#). A subset of data was created for sites that presented complete attributes of anthropogenic activity and environmental variables (Suppl. material 1) to perform the canonical correspondence analysis (CCA) ordination in R programming language v. 4.0.4 (R Core Team 2023), specifically with the vegan package (Oksanen et al. 2020). The criterion of presence and absence of *A.spicifera* in the sampling sites was evaluated through the matrices of anthropogenic activity (variables: none, NPA, tourism, fishing, nautical, runoff, mining, habitat modification and industry) and environmental factors (variables: SST_IS, Chlo_SAT, PAR_SAT, PIC_SAT, POC_SAT and SST_SAT). The contingency table of raw data was implemented in the CCA. The variance explained by the model (total inertia) and the proportion explained by the environmental variables (constrained proportion) was taken as reference. The statistical attributes of redundancy of the environmental variables were verified on the constrained variables through correlation tests (R^2^ < 0.70) and Variance Inflation Factors (around one). Additionally, statistical significance was confirmed through the permutational ANOVA test (alpha significance level < 0.05 and 999 permutations). The variables that did not match the criteria were purged and the CCA was again estimated with their respective statistical descriptors. The results were plotted in a triplot.

## Results

### Acanthophoraspicifera records

The search in Google Scholar for *A.spicifera* in La Paz Bay resulted in 109 publications. Once duplicate literature and non-scientific articles were excluded, ten papers with records of *A.spicifera* were found. Epibionts resulted in 36 articles, of which 14 were to Hydrozoa and 22 to Bryozoa. However, only one article mentions epibionts growing on this macroalga (cf. Méndez-Trejo et al. (2014)).

We compiled 709 entries (presence and absence of *A.spicifera* thalli) from 2004 to 2023 in the database (Mendoza-Becerril et al. 2023), of which 73 were obtained from literature, three from citizen science and 633 from the field. During this period, 21 sites were mentioned in literature records, three were from citizen science and 31 were visited for this study. The *A.spicifera* thalli sizes ranged from 0.7 to 15.4 cm (Table 1) and no reproductive thalli were observed in any of the cases.

**Figure 3.**
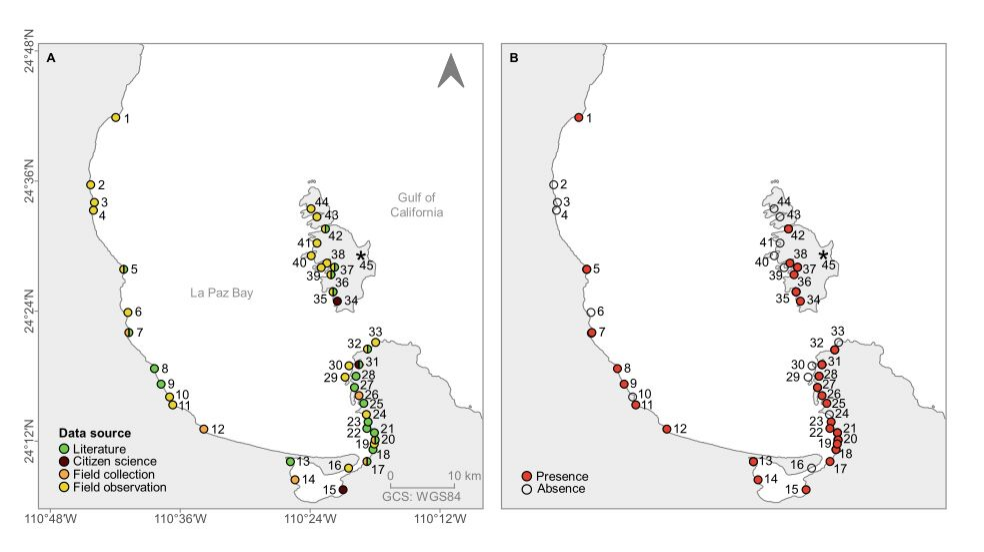
Distribution of *Acanthophoraspicifera* in La Paz Bay: **A** Data source: literature, citizen science and field sampling; **B** Presence and absence. Details of the sites are available in Table 1.

**Figure 4.**
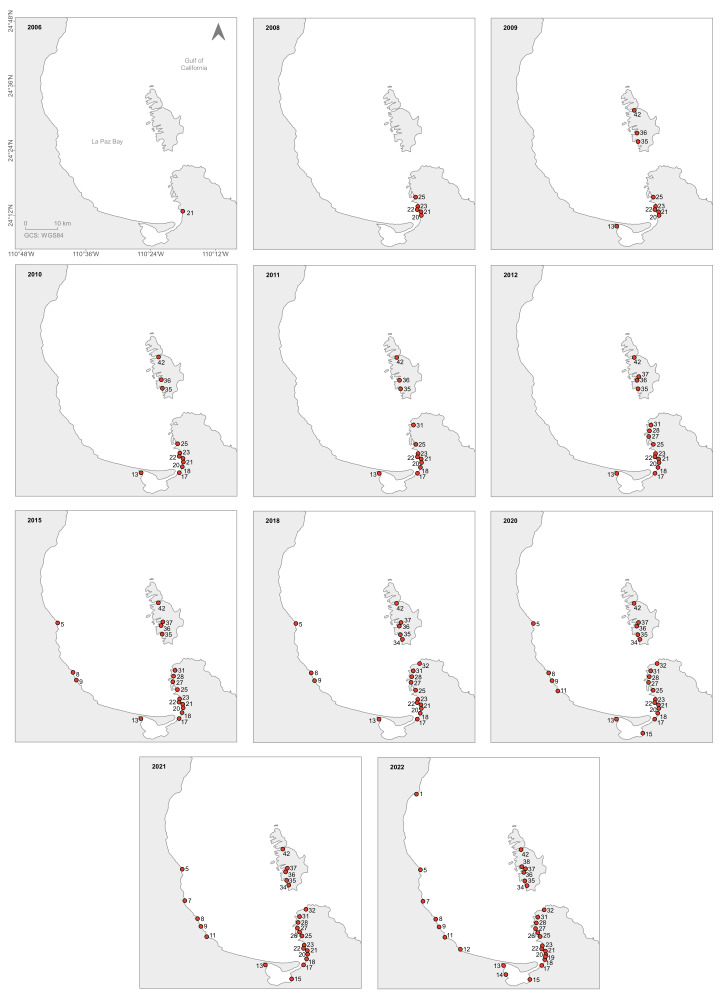
Historical records of *Acanthophoraspicifera* within the Bay of La Paz from 2006-2022. For site numbers, refer to Table 1.

It was evident that, with 31 sites visited in La Paz Bay (2020-2022), a broader view of the current distribution of *A.spicifera* was achieved, moving the northern limit previously known from literature data from Ensenada de la Partida (42) to Punta Coyote (1). Based on citizen-science data, the southern boundary previously known from literature data was also extended from Estero Zacatecas (13) to Grand Plaza harbour (15) (Fig. 3A). The distribution of *A.spicifera* in La Paz Bay is discontinuous, with the absence of this macroalga in 15 visited sites, ten from La Paz Bay and five in the ESA (Fig. 3B). The presence of new sites along the coast is shown in Fig. 4. The first record of *A.spicifera* was 2006 from Costa Baja (21). From 2008 to 2011, the species was only recorded from the south of the Bay. During 2008, it was observed in four more sites: Punta Roca Caimancito (20), Punta Prieta (22), Playa Enfermería (23) and Playa Eréndira (25). Later in 2009, its presence was recorded from Estero Zacatecas (13), Bahía San Gabriel (35), Ensenada de la Gallina (36) and Ensenada de la Partida (42); boardwalk (17) and Palmira (18) in 2010; and Estero (31) in 2011. From 2012 onwards, it was detected in other locations both south and north of the Bay: in 2012, in Playa Pichilingue (27), Estero el Merito (28) and Ensenada del Gallo (37); in 2015, in Punta Tarabillas (5), Punta de Yepiz (9) and Boca del Sauzoso (8); in 2018, in Ensenada de la Dispensa (34) and Playa el Tecolote (32) and Ensenada de la Dispensa (34); in 2020, in El Cajete (11) and Marina Grand Plaza (15); 2021 in San Juan de la Costa (7) and (UABCS Pier (26)) and 2022 in Punta Coyote (1), Punta El León (12), in front of CIBNOR (14), Playa Coromuel (19) and Ensenada La Raza (38) (Fig. 4).

### Sessile colonial epibionts

**Figure 5.**
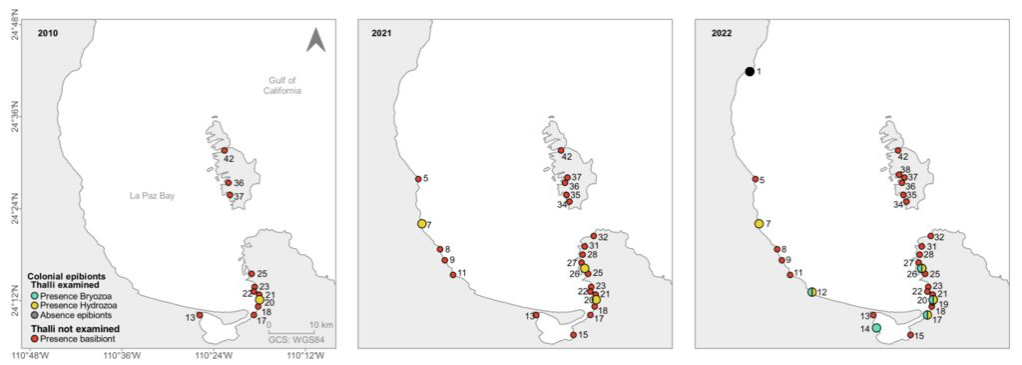
Records of colonial epibionts of *Acanthophoraspicifera* within the La Paz Bay. Colonial epibiont record type: the presence of Bryozoa (aqua), presence of Hydrozoa (yellow), absence of epibionts (grey) and the presence of basibionts without epibiont data (red). For site numbers, refer to Table 1.

**Figure 6.**
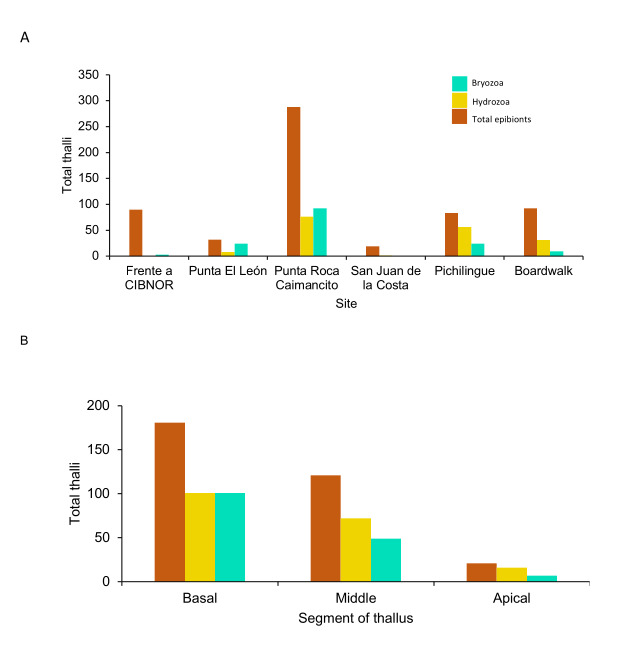
Thalli with colonial epibionts: **A** total number of thalli with colonial epibionts; **B** thallus segments with epibionts.

The first record of the presence of epibionts (Hydrozoa) was recorded in Punta Roca Caimancito (20) in 2010 (Méndez-Trejo et al. 2014; Fig. 5). After this first record of Hydrozoa, epibionts were studied in two more sites (San Juan de la Costa (7) and Pichilingue UABCS Pier (26). In addition, in 2022, in three other sites, both bryozoans and hydrozoans were recorded [(Punta El León (12), in front of CIBNOR (14) and boardwalk (17)]. In the same year, the absence of epibionts was also recorded in Punta Coyote (1) (Fig. 5). Therefore, fieldwork from this study contributed to recent accurate data on colonial epibionts of *A.spicifera* (2021-2022). Epibionts were present in six sites, but only in the boardwalk (17), Pichilingue (UABCS Pier; 26), Punta El León and Punta Roca Caimancito (20), where both bryozoans and hydrozoans were recorded throughout the thallus. The hydrozoans were present in 172 thalli and bryozoans in 152, with a heterogeneous distribution amongst the sites (Fig. 6A). Both epibiont groups predominated in the basal segment of macroalgae (201 thalli). The apical segment had the lowest epibionts record (23 thalli) (Fig. 6B).

**Figure 7.**
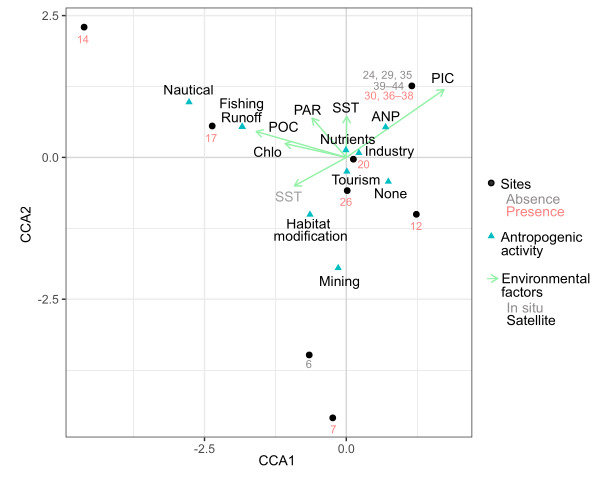
Canonical correspondence analysis (CCA) triplot for *Acanthophoraspicifera* attributes on La Paz Bay. Sites (black points); anthropogenic activities (blue triangles); absent (grey letters) and presence (red letters); and environmental factors (green arrows); in situ (grey letters) and satellite (black letters), chlorophyll-*a* (Chlo), photosynthetically available radiation (PAR), particulate inorganic carbon (PIC), particulate organic carbon (POC) and sea surface temperature (SST). Site numbers refer to Table 1.

In La Paz Bay, *A.spicifera* had environmental preferences for the depth (0–10 m), salinity (34.00–37.00 ppt), SST (22.00–29.00 °C), Chlo-a (0.24–3.22 mg m^-3^), PAR (41.68–62.58 Einstein m^-2^ d^-1^), PIC (< 0.004 mol m^-3^), POC (66.60–1,028.83 mg m^-3^) and SST (21.20–32.12 °C). Regarding anthropogenic activity, there was a higher incidence in sites with the presence of tourism (10 records), nutrient supply (nine records) and industry (eight records). The model implemented in CCA (Fig. 7) with anthropogenic activity and environmental variables did not present statistical differences (p = 0.071) and the explained variance of the raw data was 29.67%. The variables Chlo-*a*, POC and SST did not meet the redundancy criteria, the filtered contingency table being made up of the PIC and PAR variables, which did not present statistical significance in the model (p = 0.124) and the model explained 89.21%.

## Discussion

### Presence and dispersion of Acanthophora

*Acanthophoraspicifera* has not been reported along the Pacific coast in the last 159 years, based on earlier studies on Mexican algae (Agardh 1847) and posterior samplings during the twentieth century (e.g. Dawson (1944), Dawson (1966), González-González et al. (1996), Riosmena-Rodríguez and Paul-Chávez (1997)). At the end of 2022, it could be found in 30 sites along the Bay (Fig. 4), including those in ESA and some places with considerable coverage, such as in Punta Roca Caimancito (20), where, in 2009, it occupied 3.2 hectares (Schnoller et al. 2016). The records have increased chiefly since 2020 when this study started (Fig. 4). Nevertheless, the question remains as to whether these records are due to incomplete floristic studies in the past, showing that it is necessary to establish a programme with systematic observations that give us an idea about the colonisation rates along the coast and on a long timescale. As far as we know, there is a northern presence record outside the Bay in Isla San José (Riosmena-Rodríguez et al. 2014). Some of our observations that covered absent records on the outside of the Bay with the sites of El Pulguero (24°21’10.7”N, 110°13’40.7”W), Playa Caleritas (24°21’17.6”N, 110°17’04.0”W) and Bahía de los Sueños (23°59’29.8”N 109°49’48.6”W) indicate that Playa Tecolote is the southern limit of distribution at the time. It seems that *A.spicifera* prefers sandy and shallow habitats with high temperatures and slow water motion (Riosmena-Rodríguez et al. 2014). The flow fields in ocean currents could be a determinant to constrain population sizes and their distributional ranges (Gaylord and Gaines 2000). Speed is a determinant that allows the establishment of new individuals in benthic environments and their successful lives over time, so speeds more than 1 cm/s seem to affect their distribution (Gaylord et al. 2002). We think that quiescent waters inside the Bay have favoured the establishment of populations even though the occurrence mentioned above could indicate its spread to the north and south. Nevertheless, distributional limits in many seaweeds cluster at particular shoreline locations (Gaylord and Gaines 2000). The movement of this species and the presence of depths of more than 200 m in the northern portion, reaching 400 m in Alfonso Basin (Zaytsev et al. 2010) suggest that its displacement has been step-by-step over the coastline preferring, as mentioned, the shallow and sandy areas. The speed of surface currents in the northern portion called Boca Grande of up to 50 m/s (Torres-Hernández et al. 2022) may have prevented, as mentioned above, the movement and colonisation of certain bay sectors. The flow of nutrients closest to the coast where *A.spicifera* growth, such as phosphorus (Antonio-Robles et al. 2021), can also be influenced by local wind, tide, shape of the shore and, as we saw, surface current intensities.

Besides, long-distance dispersal is not the rule in marine macroalgae, not just for these oceanographic barriers, but also due to different biological limitations. The number of cosmopolitan species in seaweeds, confirmed by molecular tools, does not support this hypothesis of long-distance dispersal (Vieira et al. 2017). Although studies have shown that some species with buoyancy capacity could travel as fast as 37 km/yr, this depends on the current’s speed and direction. On the east coast of Canada, there is evidence that fragments of the green alga *Codium* could travel for three weeks (Gagnon et al. 2015b). In contrast, non-buoyant fragments dispersed over much shorter distances decreased their capacity to between 3 to 6 days after pieces of the thalli were released to the water column (Gagnon et al. 2011) and most had stopped dispersing completely (i.e. had settled to the bottom) in less than one hour (Gagnon et al. 2015a). Rafting is regarded to be an important dispersal mechanism in the marine environment, but its success largely depends on the quality of the floating substrate, particularly in seaweeds; the seawater temperatures are critical for vegetative tissue survival floating in the water, mainly in higher latitudes where 18°C seems to limit the success in dispersion (Vandendriessche et al. 2007). In filamentous algae, sediment quantities could be the factor of a rapid degradation process when these thalli are travelling along the currents, a process that is also strongly temperature-dependent (Salovius and Bonsdorff 2004).

In their phenology, biomass, and reproductive aspects, *A.spicifera* showed that the main mechanism for dispersal was thalli fragmentation since few individuals with tetraspores and only empty cystocarps were observed (Schnoller et al. 2016). No reproductive structures were seen during the present study, a three-year period, which confirms the hypothesis of Schnoller et al. (2016). For the invasive red algae, *Kappaphycus* spp., in Hawaii, vegetative propagules were the primary means of reproduction (sexual reproduction has not been observed), and its rapid spread was related to its ability to re-grow from residual tissue left at attachment points, even after removing all algal material visible to the naked eye (Conklin and Smith 2005).

### Acanthophoraspicifera and its alien status

This red macroalga has been considered an invasive alga in Mexico by various authors (Ávila et al. 2012, Méndez-Trejo et al. 2014, Riosmena-Rodríguez et al. 2014, Schnoller et al. 2016); however, this term has different interpretations: academic interpretations, based on biological properties (Richardson et al. 2000) or pragmatic or political interpretations, which involve environmental affectation (Blackburn et al. 2011, Iannone et al. 2020). According to IUCN (2023), “Invasive alien species are animals, plants, or other organisms introduced by humans, intentionally or accidentally, into places outside their natural range, negatively impacting native biodiversity, ecosystem services, or human economy and well-being”. However, according to our results and based on Richardson et al. 2000 and Blackburn et al. 2011, as key references for alien species classification, we propose a change of status for *A.spicifera*, which means that this species should be treated as a non-native species, but naturalised “Alien plants that reproduce consistently and sustain populations over many life cycles without direct intervention by humans; they often recruit offspring freely, usually close to adult plants” (Richardson et al. 2000) and in category D2 “self-sustaining population in the wild, with individuals surviving and reproducing a significant distance from the original point of introduction” (Blackburn et al. 2011). Even so, we did not find reproductive structures. It seems that morphological fragments are the vectors that colonise other areas similar to abiotic composition. *Acanthophoraspicifera* has crossed geographical, survival, reproductive and dispersal barriers and should be monitored to evaluate negative impacts on the structure or community composition. At the moment of this study, we did not see or record evidence of a negative impact on native biodiversity, ecosystem services, human economy and well-being. However, it has been mentioned that high densities of these algae could affect corals (Tsuda et al. 2008, Schnoller et al. 2016) or sponges (Ávila et al. 2012) and displace some other macroalgae (Russell 1992). For example, in a study prior to the first record of *A.spicfera* in Bahía de La Paz, *Sargassumhorridum* (as *S.sinicola*), *Spyridiafilamentosa*, *Caulerpasertularioides* and *Laurenciajohnstonii* were the most abundant species along the Bay (Casas-Valdez et al. 1997) and, recently, *A.spicifera*, along with the green alga *Caulerpaverticillata* and the red alga *S.filamentosa* were recorded as bloom-formers in La Paz Bay, whose presence coincides with genera previously reported as bloom-formers in nutrient-rich coastal waters and no changes in the algal communities were reported (Piñón-Gimate et al. 2022).

In general, the appreciation of impact is subjective because an ecological impact must be a measurable change in the environment, involving species or ecosystem and this must consider individuals, populations, communities, environment and space (Ricciardi et al. 2013). The fact that a species is foreign has an impact; this impact could be positive or negative and the effects should be compared across time and space. Most introductions do not have studies in this sense (Russell 1992, Parker et al. 1999).

The results of CCA (Fig. 7) suggest the need to strengthen the investigation of *A.spicifera* to monitor the ecological progress of the expansion process, understand the environmental preferences at annual and interannual scales and, finally, the epiphytes’ role in La Paz Bay biodiversity. Considering that the SST has been an explanatory variable in the response to its non-native distribution in the Pacific Ocean (Camacho and Houk 2020, Chávez-Sánchez et al. 2022, Ward et al. 2023), as well as the presence of precipitation that favours its distribution in both native Atlantic (Kilar and McLachlan 1986) and non-native Pacific areas (Camacho and Houk 2020), which it is limited by the aridity of La Paz Bay (Obeso-Nieblas et al. 2004), however, could be compensated by anthropogenic nutrient inputs in the area and the high capacity of this macroalgae to absorb nutrients (Dulai et al. 2021).

### Epibionts as a side effect in the process of colonisation

Epibionts, mainly as epiphytes, amongst seaweeds, have been studied long ago in Mexico. In the beginning, only names of species growing over other thalli were recorded (Huerta Muzquiz and Tirado 1970), later with specific information about what species over which one is growing (Mateo-Cid and Mendoza-González 2012). However, the presence of colonial epibionts of *A.spicifera* was published ten years ago for the first time (Méndez-Trejo et al. 2014). In *A.spicifera*, we evaluated the presence of Bryozoa and Hydrozoa in six localities along the Bay shoreline, which we detected without a clear dominance of one of them (Fig. 6A). However, Bryozoa were present in more thalli than Hydrozoa in Punta El León (12) and Punta Roca Caimancito (20); meanwhile, Hydrozoa was the main group in Pichillingue (26) and boardwalk (17). These four localities are protected areas without significant water currents compared with open areas like San Juan de la Costa (7) due to dominant winds that blow from the east all year around and keep the southern side of the Bay protected from heavy currents (Turrent and Zaitsev 2014). They also maintain lower temperatures than the northern parts of the Bay (Herrera Cervantes 2019). Therefore, it is suggested that currents and temperature patterns can favour the greatest number of thalli (Table 1) and their epibionts: Hydrozoa and Bryozoa.

The presence of epibionts related to their position in the thallus (Fig. 6B) could be associated with how long the process of the establishment has occurred since they arrived at *A.spicifera* so that young thalli could present epibionts mainly at the base and the older ones in the three sections or it could be that lower sections are closer to the substratum and, therefore, protected from water movements when compared to the upper sections, also preventing the detachment of the thalli. As hypothesised in macroalgae of the genus *Sargassum*, the early colonisers, such as hydroids, are located in the basal part of the thallus, while late epibionts, such as bryozoans, tend to occur in apical portions (cf. Rackley (1974)). Nevertheless, experimental work is necessary to answer these questions.

Seaweeds as floating rafts have been studied recently. Kim et al. (2019) found 185 species of epibionts travelling on *Sargassumhorneri* (Turner) C. Agardh; from those, 23% were Opisthokonts, where bryozoans were one of the ten taxa recorded. Mendoza-Becerril et al. (2020) recorded 14 taxa of Hydrozoa as epibionts of the two rafting species, *S.fluitans* (Børgesen) Børgesen and *S.natans* (Linnaeus) Gaillon and one benthic species, S.polyceratiumvar.ovatum (Collins) W. R. Taylor. *Sargassum* thalli provided the substrate, food source and refuge to these organisms, which could be transferred to new places and habitats (Kim et al. 2019). A high diversity of epibionts has also been observed in both native and non-native macroalgae; however, the invasive macroalga *Undariapinnatifida* supports an impoverished or distinct epibiont assemblage related to the differences in life history between it and the native kelps, differences in biogenic habitat structure and growth strategies (Smale et al. 2013,Arnold et al. 2015).

## Conclusions

*Acanthophoraspicifera* has spread throughout La Paz Bay over the years since the first reports from Costa Baja in 2006. The historical presence of this macroalga in the Gulf of California represents a naturalised alien population, restricted at the moment to Bahía de La Paz, close surrounding areas and common along the intertidal coast of the Bay with nutrient supply. Systematic studies are necessary to evaluate its colonisation rates related to its precise environmental preferences, the possible effects "impacts" on the whole biota of the area and the environment through integrative analysis and its epibionts as a source of new non-native organisms representing the holobiont nature of *A.spicifera*. It is essential to answer if those Hydrozoa and Bryozoa found are part of the native fauna or if they travelled with their basibiont through taxonomic studies of epibionts that categorise their native and non-native substrates, including artificial structures.

## Supplementary Material

0981C840-F0CD-5489-8387-5984283053C1 10.3897/BDJ.11.e114262.suppl1 Supplementary material 1 Table S1 Data type Dataset used in the Canonical correspondence analysis Brief description Dataset used in the Canonical correspondence analysis for the sites with *Acanthophoraspicifera* records from La Paz Bay.In situ and satellite environmental variables [Chlo-a = Chlorophyll-*a* (mg m^-3^), PAR = Photosynthetically Available Radiation (Einstein m^-2^ d^-1^), PIC = Particulate Inorganic Carbon (mol m^-3^), POC = Particulate Organic Carbon (mg m^-3^) and Sea Surface Temperature (°C)]. Anthropogenic activity carried out (A = none, B = Natural Protected Area, C = tourism, D = fishing, E = nutrient supply, F = metal supply, G = nautical traffic, H = runoff water, I = mining, J = physical habitat modification, K = dump, L = industry and M = mangrove deforestation). File: oo_918870.docx https://binary.pensoft.net/file/918870 María A. Mendoza-Becerril, Francisco F. Pedroche, Mariae C. Estrada-González, Elisa Serviere-Zaragoza

## Figures and Tables

**Table 1.** Records of *Acanthophoraspicifera* and its colonial epibionts from La Paz Bay. Presence and absence of thalli, considering all data sources [LT(#) = literature (studies), CS = citizen science, FO = field observation, FC = field collection]. Thalli segments with epibionts (basal, middle, apical, ne = no epibionts, nd = no data). Type of colonial epibionts (B = Bryozoa, H = Hydrozoa). No data (nd). *Sites without specific coordinates. – non-applicable. (1) Muñoz-Ochoa et al. (2010); (2) Ávila et al. (2012); (3) Méndez-Trejo et al. (2014); (4) Riosmena-Rodríguez et al. (2014); (5) López-Vivas et al. (2016); (6) Schnoller et al. (2016); (7) Tenorio-Rodriguez et al. (2017); (8) Di Filippo-Herrera et al. (2019); (9) Schnoller et al. (2020); (10) Antonio-Robles et al. (2021); *Hernández Carmona pers. comm.; ^+^López-Vivas pers. comm. correction from locality Punta Piedra to Punta Prieta.

**ID site**	**Site**	**Date (this study)**	**Thalli of *Acanthophoraspicifera***						
			**Presence**	**Absence**	**Average size (cm)**	**Examined (number)**	**Segment with epibionts**		
							**Basal**	**Middle**	**Apical**
1	Punta Coyote	2022	FO	–	–	–	ne	ne	ne
2	Punta El Cobre	2021	–	FO	–	–	nd	nd	nd
						
3	Punta Las Ánimas	2021	–	FO	–	–	nd	nd	nd
4	Punta El Guano	2021	–	FO	–	–	nd	nd	nd
5	Punta Tarabillas	2021	LT (8*)	FO	–	–	nd	nd	nd
6	ROFOMEX San Juan de la Costa	2021, 2022		FO	–	–	nd	nd	nd
7	San Juan de la Costa	2021, 2022	FC, LT (10)	–	12.1 ± 3.3	19	H	H	nd
8	Boca del Sausozo	–	LT (8*)	–	–	–	nd	nd	nd
9	Punta de Yepiz	–	LT (8*)	–	–	–	nd	nd	nd
10	Califín	2021	–	FO	–	–	nd	nd	nd
11	El Cajete	2020	FO	–	–	–	ne	ne	ne
12	Punta El León	2022	FC	–	9.7 ± 2.8	32	B, H	B, H	B, H
13	Estero Zacatecas		LT (5)	–	–	–	nd	nd	nd
14	In front CIBNOR	2022	FC	–	6.3 ± 2.9	90	B	ne	ne
15	Grand Plaza harbor	–	CS	–	–	–	nd	nd	nd
16	Mogote Pier	2022	–	FO	–	–	nd	nd	nd
17	Boardwalk	2022	FC; LT (5,10)	–	6.4 ± 1.9	92	B, H	B, H	H
18	Palmira		LT(5)	–	–	–	nd	nd	nd
19	Playa Coromuel	2022	FO	–	–	–	nd	nd	nd
20	Punta Roca Caimancito (La Concha)	2021, 2022	FC, LT (2, 3, 4, 5, 6, 9)	–	5.8 ± 5.3	305	B, H	B, H	B, H
21	Costa Baja	–	LT (1, 4, 5, 7)	–	–	–	nd	nd	nd
22	Punta Prieta	–	LT (4^+^)	–	–	–	nd	nd	nd
23	Playa Enfermería	–	LT (4)	–	–	–	nd	nd	nd
24	Playa Sola	2022		FO	–	–	nd	nd	nd
25	Playa Eréndira	–	LT (4)	–	–	–	nd	nd	nd
26	Pichilingue (UABCS Pier)	2021, 2022	FC	–	9.5 ± 16.3	83	B, H	B, H	B, H
27	Playa Pichilingue	–	LT (5)	–	–	–	nd	nd	nd
28	Balandra Bay, Estero el Merito	–	LT (5)	–	–	–	nd	nd	nd
29	Isla San Rafaelito	2022	–	FO	–	–	nd	nd	nd
30	Punta el Diablo	2021, 2022	–	FO	–	–	nd	nd	nd
31	Balandra Bay, Estero Balandra	–	CS; LT (5)	–	–	–	nd	nd	nd
32	Playa el Tecolote	2022	LT (10)	FO	–	–	ne	ne	ne
33	Punta las Pilitas	2022	–	FO	–	–	nd	nd	nd
34	Ensenada de Dispensa	–	CS	–	–	–	nd	nd	nd
35	Bahía San Gabriel	2022	FO, LT (5)	–	–	–	nd	nd	nd
36	Ensenada de la Gallina	2022	FO, LT (5)	–	–	–	nd	nd	nd
37	Ensenada del Gallo	2022	FO, LT (5)	–	–	–	nd	nd	nd
38	Ensenada la Raza	2022	FO	–	–	–	nd	nd	nd
39	Isla Gallo	2022	–	FO	–	–	nd	nd	nd
40	Isla Ballena	2022	–	FO	–	–	nd	nd	nd
41	Ensenada del Candelero	2022	–	FO	–	–	nd	nd	nd
42	Ensenada de la Partida	2022	LT (5)	FO	–	–	nd	nd	nd
43	Ensenada el Cardonal	2022	–	FO	–	–	nd	nd	nd
44	Ensenada Grande	2022	–	FO	–	–	nd	nd	nd
45	Espíritu Santo Island*	–	LT (4)	–	–	–	nd	nd	nd
