# Supplementary material for: Records of the non‐native alga Acanthophoraspicifera (Rhodophyta) and their colonial epibionts in La Paz Bay, Gulf of California
Source: Biodivers Data J. 2023 Nov 20;11:e114262. doi: 10.3897/BDJ.11.e114262 (PMC10682994; doi:10.3897/BDJ.11.e114262)
Supplement: Supplementary material 1 — Table S1 [file bdj-11-e114262-s001.docx]

Table S1. Dataset used in the Canonical correspondence analysis for the sites with *Acanthophora spicifera* records from La Paz Bay. In situ and satellite environmental variables [Chlo-a = Chlorophyll-*a* (mg m^-3^), PAR = Photosynthetically Available Radiation (Einstein m^-2^ d^-1^), PIC = Particulate Inorganic Carbon (mol m^-3^), POC = Particulate Organic Carbon (mg m^-3^) and Sea Surface Temperature (°C)]. Anthropogenic activity carried out (A = none, B = Natural Protected Area, C = tourism, D = fishing, E = nutrient supply, F = metal supply, G = nautical traffic, H = runoff water, I = mining, J = physical habitat modification, K = dump, L = industry and M = mangrove deforestation).

| **ID site** | **Site** | **Record** | **In situ** | **Satellite** | | | | | **Anthropogenic activity** | | | | | | | | | | | | |
| --- | --- | --- | --- | --- | --- | --- | --- | --- | --- | --- | --- | --- | --- | --- | --- | --- | --- | --- | --- | --- | --- |
|  |  |  | **SST** | **Chlo-*a*** | **PAR** | **PIC** | **POC** | **SST** | **A** | **B** | **C** | **D** | **E** | **F** | **G** | **H** | **I** | **J** | **K** | **L** | **M** |
| 6 | ROFOMEX San Juan de la Costa | Absence | 27.170 | 0.270 | 36.872 | 0.000 | 65.799 | 27.258 | 0 | 0 | 0 | 0 | 0 | 1 | 0 | 0 | 1 | 1 | 0 | 0 | 0 |
| 6 | ROFOMEX San Juan de la Costa | Absence | 27.170 | 1.853 | 62.050 | 0.000 | 299.200 | 25.995 | 0 | 0 | 0 | 0 | 0 | 1 | 0 | 0 | 1 | 1 | 0 | 0 | 0 |
| 7 | San Juan de la Costa | Prescence | 27.000 | 2.261 | 58.638 | 0.000 | 300.360 | 23.790 | 0 | 0 | 0 | 0 | 0 | 1 | 0 | 0 | 1 | 0 | 0 | 0 | 0 |
| 12 | Punta León | Prescence | 26.000 | 1.032 | 62.588 | 0.000 | 152.400 | 26.535 | 1 | 0 | 0 | 0 | 0 | 0 | 0 | 0 | 0 | 0 | 0 | 0 | 0 |
| 14 | Frente a CIBNOR | Prescence | 28.500 | 3.226 | 61.152 | 0.000 | 1028.838 | 27.365 | 0 | 0 | 0 | 0 | 0 | 0 | 1 | 0 | 0 | 0 | 0 | 0 | 0 |
| 17 | Casa del Marino | Prescence | 27.000 | 2.341 | 61.682 | 0.000 | 519.200 | 27.256 | 0 | 0 | 0 | 1 | 1 | 0 | 1 | 0 | 0 | 1 | 0 | 0 | 0 |
| 20 | Punta Roca Caimancito (La Concha) | Prescence | 28.600 | 1.074 | 43.840 | 0.000 | 224.000 | 29.143 | 0 | 0 | 1 | 0 | 1 | 0 | 0 | 0 | 0 | 0 | 0 | 1 | 0 |
| 20 | Punta Roca Caimancito (La Concha) | Prescence | 22.000 | 1.168 | 48.382 | 0.001 | 298.000 | 23.025 | 0 | 0 | 1 | 0 | 1 | 0 | 0 | 0 | 0 | 0 | 0 | 1 | 0 |
| 20 | Punta Roca Caimancito (La Concha) | Prescence | 22.000 | 1.551 | 53.686 | 0.000 | 341.945 | 22.128 | 0 | 0 | 1 | 0 | 1 | 0 | 0 | 0 | 0 | 0 | 0 | 1 | 0 |
| 20 | Punta Roca Caimancito (La Concha) | Prescence | 23.000 | 1.235 | 58.410 | 0.001 | 205.600 | 23.630 | 0 | 0 | 1 | 0 | 1 | 0 | 0 | 0 | 0 | 0 | 0 | 1 | 0 |
| 20 | Punta Roca Caimancito (La Concha) | Prescence | 24.000 | 1.214 | 61.438 | 0.000 | 179.341 | 25.450 | 0 | 0 | 1 | 0 | 1 | 0 | 0 | 0 | 0 | 0 | 0 | 1 | 0 |
| 20 | Punta Roca Caimancito (La Concha) | Prescence | 29.000 | 0.248 | 55.968 | 0.000 | 66.600 | 32.125 | 0 | 0 | 1 | 0 | 1 | 0 | 0 | 0 | 0 | 0 | 0 | 1 | 0 |
| 20 | Punta Roca Caimancito (La Concha) | Prescence | 25.500 | 2.561 | 41.686 | 0.004 | 451.200 | 27.115 | 0 | 0 | 1 | 0 | 1 | 0 | 0 | 0 | 0 | 0 | 0 | 1 | 0 |
| 20 | Punta Roca Caimancito (La Concha) | Prescence | 24.000 | 0.708 | 60.724 | 0.000 | 127.800 | 25.590 | 0 | 0 | 1 | 0 | 1 | 0 | 0 | 0 | 0 | 0 | 0 | 1 | 0 |
| 24 | Playa Sola | Absence | 24.000 | 24.439 | 61.838 | 0.000 | 1784.200 | 23.479 | 0 | 1 | 0 | 0 | 0 | 0 | 0 | 0 | 0 | 0 | 0 | 0 | 0 |
| 26 | Muelle UABCS Pichilingue | Prescence | 25.000 | 2.032 | 42.402 | 0.000 | 359.200 | 21.205 | 0 | 0 | 1 | 0 | 0 | 0 | 0 | 0 | 0 | 0 | 0 | 0 | 0 |
| 26 | Muelle UABCS Pichilingue | Prescence | 27.800 | 0.541 | 61.578 | 0.000 | 140.400 | 28.153 | 0 | 0 | 1 | 0 | 0 | 0 | 0 | 0 | 0 | 0 | 0 | 0 | 0 |
| 29 | La Lobera San Rafaelito | Absence | 23.000 | 1.350 | 61.980 | 0.000 | 237.200 | 27.535 | 0 | 1 | 0 | 0 | 0 | 0 | 0 | 0 | 0 | 0 | 0 | 0 | 0 |
| 30 | Punta Diablo | Prescence | 23.000 | 1.350 | 61.980 | 0.000 | 237.200 | 27.535 | 0 | 1 | 0 | 0 | 0 | 0 | 0 | 0 | 0 | 0 | 0 | 0 | 0 |
| 35 | Ensenada San Gabriel coral | Absence | 25.500 | 0.706 | 56.954 | 0.003 | 138.412 | 27.509 | 0 | 1 | 0 | 0 | 0 | 0 | 0 | 0 | 0 | 0 | 0 | 0 | 0 |
| 36 | Ensenada La Gallina | Prescence | 27.500 | 0.706 | 56.954 | 0.003 | 138.412 | 27.509 | 0 | 1 | 0 | 0 | 0 | 0 | 0 | 0 | 0 | 0 | 0 | 0 | 0 |
| 37 | Ensenada El Gallo | Prescence | 27.000 | 0.615 | 50.462 | 0.002 | 120.809 | 27.662 | 0 | 1 | 0 | 0 | 0 | 0 | 0 | 0 | 0 | 0 | 0 | 0 | 0 |
| 38 | Ensenada La Raza | Prescence | 26.500 | 0.615 | 50.462 | 0.002 | 120.809 | 27.662 | 0 | 1 | 0 | 0 | 0 | 0 | 0 | 0 | 0 | 0 | 0 | 0 | 0 |
| 39 | Isla El Gallo | Absence | 28.500 | 0.630 | 49.860 | 0.002 | 123.271 | 27.685 | 0 | 1 | 0 | 0 | 0 | 0 | 0 | 0 | 0 | 0 | 0 | 0 | 0 |
| 40 | Isla Ballena | Absence | 25.500 | 0.630 | 49.860 | 0.002 | 123.271 | 27.685 | 0 | 1 | 0 | 0 | 0 | 0 | 0 | 0 | 0 | 0 | 0 | 0 | 0 |
| 41 | El Candelero | Absence | 25.500 | 0.514 | 47.462 | 0.001 | 110.143 | 28.413 | 0 | 1 | 0 | 0 | 0 | 0 | 0 | 0 | 0 | 0 | 0 | 0 | 0 |
| 42 | Ensenada La Partida | Absence | 26.000 | 0.514 | 47.462 | 0.001 | 110.143 | 28.413 | 0 | 1 | 0 | 0 | 0 | 0 | 0 | 0 | 0 | 0 | 0 | 0 | 0 |
| 43 | El Cardonal | Absence | 25.000 | 0.435 | 56.112 | 0.001 | 101.828 | 28.413 | 0 | 1 | 0 | 0 | 0 | 0 | 0 | 0 | 0 | 0 | 0 | 0 | 0 |
| 44 | Ensenada Grande | Absence | 24.000 | 0.435 | 56.112 | 0.001 | 101.828 | 28.413 | 0 | 1 | 0 | 0 | 0 | 0 | 0 | 0 | 0 | 0 | 0 | 0 | 0 |
